# The Role of Nitrogen‐doping in the Catalytic Transfer Hydrogenation of Phenol to Cyclohexanone with Formic Acid over Pd supported on Carbon Nanotubes

**DOI:** 10.1002/chem.202100981

**Published:** 2021-06-18

**Authors:** Bin Hu, Xiaoyu Li, Wilma Busser, Stefan Schmidt, Wei Xia, Guangci Li, Xuebing Li, Baoxiang Peng

**Affiliations:** ^1^ Laboratory of Industrial Chemistry Ruhr University Bochum Universitätsstraße 150 44780 Bochum Germany; ^2^ Max Planck Institute for Chemical Energy Conversion Stiftstraße 34–36 45470 Mülheim a. d. Ruhr Germany; ^3^ Qingdao Institute of Bioenergy and Bioprocess Technology Chinese Academy of Sciences 266101 Qingdao China

**Keywords:** Deactivation, formic acid decomposition, N-doping, nonplanar adsorption, transfer hydrogenation

## Abstract

Highly selective one‐step hydrogenation of phenol to cyclohexanone, an important intermediate in the production of nylon 6 and nylon 66, is desirable but remains a challenge. Pd nanoparticles supported on nitrogen‐ and oxygen‐functionalized carbon nanotubes (NCNTs, OCNTs) were prepared, characterized, and applied in the hydrogenation of phenol to cyclohexanone to study the effect of N‐doping. Almost full conversion of phenol with high selectivity to cyclohexanone was achieved over Pd/NCNT under mild reaction conditions using either H_2_ or formic acid (FA) as a hydrogen source. The effects of reaction temperature and FA/phenol ratio and the reusability were investigated. Separate FA decomposition experiments without and with the addition of phenol were performed to investigate the reaction mechanism, especially the deactivation behavior. Deactivation was observed for both catalysts during the FA decomposition, while only Pd/OCNT rather than Pd/NCNT was deactivated in the transfer hydrogenation with FA and the FA decomposition in the presence of phenol, indicating the unique role of N‐doping. Therefore, we assume that deactivation is caused by the strongly bound formates on the active Pd sites, suppressing further FA decomposition and/or transfer hydrogenation on Pd. The nonplanar adsorption of phenol on NCNTs via weak O−H⋅⋅⋅N interactions enables the occurrence of the subsequent hydrogenation by adsorbed formate on Pd.

## Graphical abstract

**Pd/OCNT is deactivated during the transfer hydrogenation of phenol** with formic acid to cyclohexanone because of the full coverage of formate on Pd. By comparison, Pd/NCNT is highly active and selective in the transfer hydrogenation and deactivation does not occur. The nonplanar adsorption of phenol on NCNTs via weak O−H⋅⋅⋅N interactions enables the transfer hydrogenation with the adsorbed formate on Pd.

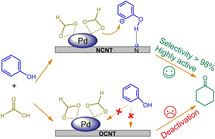

## Introduction

The utilization of renewable feedstocks, especially lignocellulosic biomass for the production of biofuels and commodity chemicals, has attracted significant and growing attention.^[1]^ Selective hydrogenation of biomass‐derived phenol to cyclohexanone has been of continuous interest.^[2]^ Cyclohexanone is of high industrial importance because of its use as an intermediate in the production of caprolactam and adipic acid, which are used to manufacture nylon 6 and nylon 66, respectively.^[3]^ Phenol can be hydrogenated to cyclohexanone in a one‐step or a two‐step process.^[4]^ The two‐step process involves the hydrogenation of phenol to cyclohexanol, followed by a subsequent dehydrogenation to cyclohexanone.^[5]^ The one‐step selective hydrogenation of phenol to cyclohexanone is certainly preferred due to avoiding the endothermic dehydrogenation of cyclohexanol. Therefore, the development of an effective catalyst for phenol hydrogenation has attracted intense research interest but remains a challenge.^[6]^

**Scheme 1:**
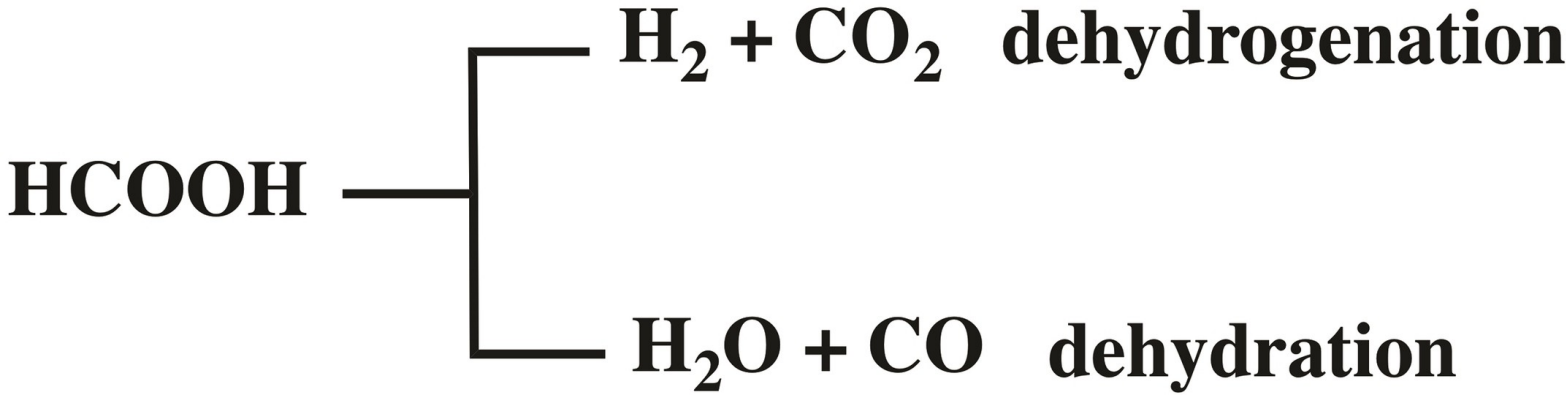
Pathways for the decomposition of FA.

Formic acid (FA), which is one of the major products formed in lignocellulosic biomass processing and also accessible via a variety of chemical processes based on the hydrolysis of methyl formate or the electrochemical reduction of CO_2_, is considered to be a renewable source for hydrogen production and a hydrogen donor for catalytic transfer hydrogenation (CTH) reactions.^[7]^ Hydrogen stored in FA can be released in situ on demand by catalytic dehydrogenation, with low levels of CO being an undesirable byproduct, which is generally produced by FA dehydration (Scheme 1).^[8]^ Pd was found to be the optimum metal for the liquid‐phase decomposition of FA.^[9]^

As a hydrogen donor, FA has been used for decades in homogeneous and heterogeneous CTH systems, including aldehydes,^[10]^ olefins,^[11]^ nitroarenes,^[12]^ and hydrodechlorination.^[13]^ Recently, FA has been widely employed as a hydrogen source in biomass conversion via catalytic transfer hydrogenation reactions.^[14]^ We reported the quantitative conversion of 5‐hydroxymethylfurfural to 2,5‐dimethylfuran with FA under mild conditions over Pd/NMC.^[15]^ The key strategy is the utilization of FA, serving multiple roles as acidic catalyst, hydrogen donor, catalyst moderator inhibiting ring hydrogenation, and shifting the dominant reaction pathway. Hann and co‐workers^[16]^ reported the hydrogenation of C_6_‐sugars to γ‐valerolactone using FA as the hydrogen donor over Ru catalysts.

Nitrogen‐doped carbon materials have been demonstrated to be promising catalyst supports.^[17]^ Doping with nitrogen atoms modifies the surface structure of carbon materials and endows enhanced basicity,^[18]^ which improves the catalytic activity of supported metal catalysts, such as Pd, Pt, and Ni.^[19]^ Antonietti and co‐workers demonstrated that Pd nanoparticles (NPs) supported on a mesoporous graphitic carbon nitride (Pd@mpg‐C_3_N_4_) are highly catalytically active, enabling the selective formation of cyclohexanone under mild conditions with H_2_.^[19b]^ Li and co‐workers reported that N‐functionalized ordered mesoporous carbon supported Pd exhibited enhanced catalytic performance and cyclohexanone selectivity in the phenol hydrogenation with H_2_.^[20]^ Furthermore, FA as the alternative hydrogen source has been reported for the catalytic phenol hydrogenation over Pd/AC.^[21]^ Sodium formate has also been utilized as in situ hydrogen source for phenol hydrogenation over Pd/C.^[22]^ Nevertheless, the role of the N‐doped carbon support in the catalytic transfer hydrogenation of phenol with FA has not been elucidated and discussed in detail.

Up to now, the investigations of catalyst deactivation during phenol hydrogenation have been performed with H_2_, showing that it is mainly caused by coke deposition.^[6a]^ Generally, the deposition of coke depends on the presence of strong acid sites. For instance, Pd/Al_2_O_3_ suffers from deactivation owing to the presence of strong acidic sites on the alumina support.^[23]^ For transfer hydrogenation of phenol with FA, the deactivation mechanism, which is expected to be different from the deactivation in H_2_, has not yet been discussed.

In the present study, we aim at developing an active, selective, and stable Pd‐based catalyst for the one‐step transfer hydrogenation of phenol with FA to cyclohexanone under mild reaction conditions in the aqueous phase. The role of N‐doping was systematically investigated by varying the catalyst support (NCNTs vs. OCNTs) and the hydrogen donor (H_2_ vs. FA). The decomposition of FA without and with the addition of phenol was performed to understand the deactivation mechanism.

## Results and Discussion

### Characterization

The physicochemical properties of all carbon supports and supported Pd catalysts are summarized in Table 1. Both CNTs show similar pore volumes and pore size distributions. OCNTs display a high specific surface area of 402 m^2^/g. After doping with N, the specific surface area of the NCNTs is slightly increased, amounting to 454 m^2^/g. The content of O‐functionalized groups on the surface of NCNTs (7.8 at%, Table 2) determined by XPS is slightly lower compared with OCNTs (8.9 at%). The major difference between OCNTs and NCNTs is the different surface chemistry due to the considerable amount of nitrogen (2.8 at%, Table 2) embedded in the surface of NCNTs. It is assumed that the surface chemistry of the support, especially the functional groups and N‐dopants, can significantly alter the dispersion and electronic properties of the supported metal nanoparticles and thus influence their catalytic activities.^[24]^ For comparison, the properties of AC and Pd/AC are also listed in Table 1.

**Table 1:** Physicochemical properties of carbon materials and supported Pd catalysts.

Sample	*M*^[a]^ [wt %]	*S_BET_*^[b]^ [m^2^g^−1^]	*V*_pore_ [cm^3^g^−1^]	*d*_pore_^[c]^ [nm]	*d*_Pd_^[d]^ [nm]
NCNT	–	454	1.0	8.9	–
OCNT	–	402	0.9	8.9	–
AC	–	617	0.5	3.3	–
Pd/NCNT	0.95	434	0.9	8.5	4.3
Pd/OCNT	0.98	399	0.9	8.9	5.1
Pd/AC	0.98	473	0.4	3.4	7.8

[a] Metal loading determined by AAS; [b] BET specific surface area; [c] Average pore diameter; [d] Mean Pd particle size determined by TEM.

**Table 2:** Chemical compositions of Pd/NCNT, Pd/OCNT and the supports.

Sample	Pd (wt/at %)			N (at %)		O (at %)
	Bulk^[a]^	surf.^[b]^		surf.^[b]^		surf.^[b]^
NCNT	–	–		2.8		7.8
OCNT	–	–		–		8.9
Pd/NCNT	0.97	1.04		3.2		8.1
Pd/OCNT	0.94	0.53		–		9.4

[a] Bulk composition determined by AAS; [b] Surface composition determined by XPS assuming a homogenous model distribution.

**Figure 1:**
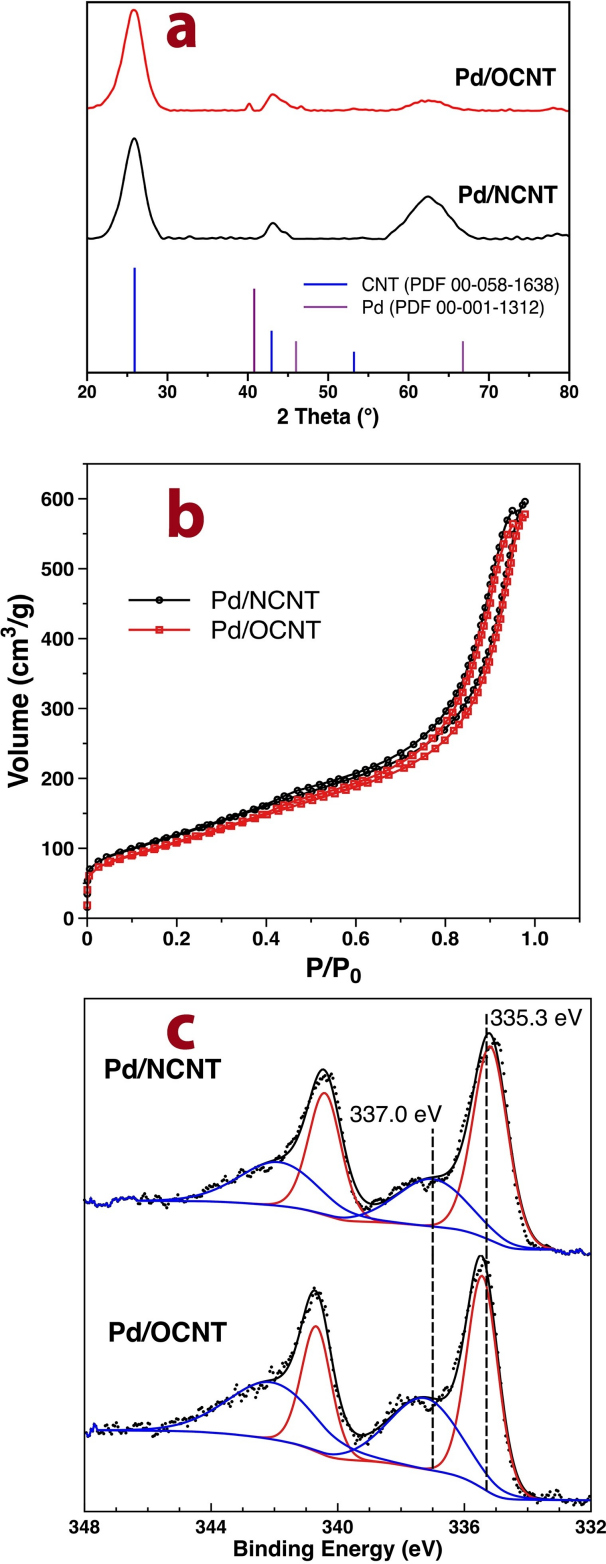
(a) XRD patterns of Pd/NCNT and Pd/OCNT (Note that the broad reflections at around 62° originate from the Si holder applied in the XRD measurements); (b) N_2_ adsorption/desorption isotherms of Pd/NCNT and Pd/OCNT; (c) Pd 3d regions of the deconvoluted XPS results for Pd/NCNT and Pd/OCNT.

**Figure 2:**
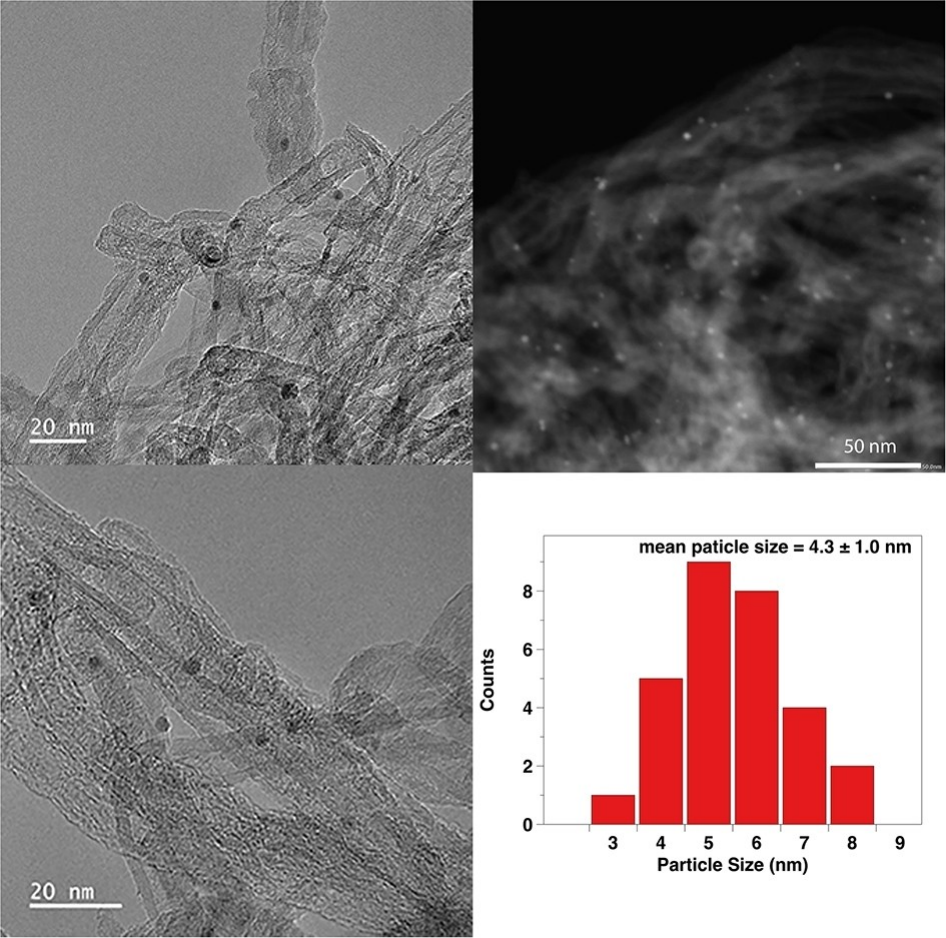
Bright‐field and dark‐field TEM images as well as particle size distribution of Pd/NCNT.

After the deposition of Pd nanoparticles, the CNTs‐supported catalysts also show the same isotherm type (Figure 1b) and pore size distribution (Table 1). The actual Pd loadings determined by AAS are comparable for all three catalysts and close to their expected values of 1 wt% (Table 1). XRD patterns of the CNT‐supported Pd samples are shown in Figure 1a. Both samples exhibit the characteristic reflections of graphitic carbon at ca. 26° and 42.4°, corresponding to the (002) and (111) planes of the hexagonal graphite structure of CNTs, respectively. Despite similar Pd loadings, Pd/OCNT shows clearly visible reflections at 40.2° and 46.7° attributed to the characteristic (111) and (200) reflections of Pd NPs (PDF card #00‐001‐1312), whereas no significant Pd reflections are identified for Pd/NCNT, suggesting a higher Pd dispersion and/or a smaller number of relatively larger Pd nanoparticles above 5 nm, which can be detected by XRD.^[25]^ Such a distinct difference was also observed for Pd supported on N‐free and N‐containing mesoporous carbon in our previous study.^[15]^ This observation is in good agreement with our previous reports, showing that N‐doped carbon and TiO_2_ can effectively enhance the dispersion of metal NPs.[^15^, ^26^] The representative TEM images and the corresponding Pd particle size distributions of Pd/NCNT and Pd/OCNT are shown in Figures 2 and S3.

EDS elemental mapping showed that the N dopants are homogeneously distributed in the NCNT, and the Pd NPs are preferably anchored on these N species (Figure S2). The mean Pd particle sizes of Pd/NCNT and Pd/OCNT are relatively similar, amounting to 4.3 and 5.1 nm, respectively, allowing a direct comparison of their catalytic performances in the hydrogenation of phenol excluding the effect of particle size.

The surface compositions of the supported Pd catalysts were examined by XPS. The survey spectra are shown in Figure S4, and the derived compositions of the Pd catalysts and the carbon materials are summarized in Table 2. The surface Pd concentration of Pd/NCNT (1.04 at%) is higher than that of Pd/OCNT (0.53 at%), indicating the positive effect of N‐doping on the dispersion of Pd NPs. In the Pd 3d_5/2_ spectra, the major Pd 3d_5/2_ peak was found to be at 335.3 eV for both catalysts, whereas a considerable fraction at 337.0 eV was also detected (Figure 1c and Table S1). The two contributions at 335.3 and 337.0 eV can be assigned to Pd^0^ and PdO, respectively.[^19d^, ^24d^] Furthermore, the surface N concentration of Pd/NCNT was determined to be 3.2 at%. The N 1s core level XP spectrum of Pd/NCNT (Figure S5, Table S2) suggests the presence of both pyridinic N (398.6 eV) and pyrrolic N (400.2 eV).

### Catalytic conversion of phenol to cyclohexanone

#### Effect of catalyst support and hydrogen sources

**Figure 3:**
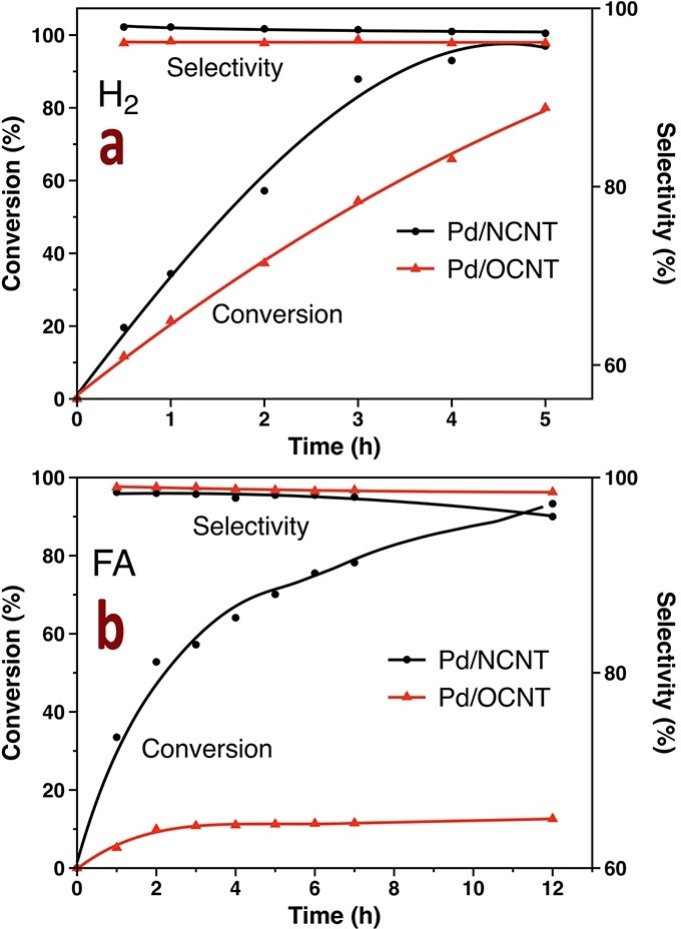
Time course of the hydrogenation of phenol to cyclohexanone over Pd/NCNT and Pd/OCNT in the presence of (a) molecular H_2_ or (b) FA. Reaction conditions: 0.05 mmol phenol, 30 mg catalyst, 60 °C, (a) 3 mmol formic acid, or (b) 1 bar H_2_.

The effect of the catalyst support on the catalytic performance in the hydrogenation of phenol to cyclohexanone was investigated using either molecular H_2_ or FA as the hydrogen source. No conversion was observed for the blank reactions without catalysts with either H_2_ or FA. When H_2_ was used, the degrees of phenol conversion after 3 h were 87.9 %, 54.4 %, and 50.2 % over Pd/NCNT, Pd/OCNT, and Pd/AC (Table 3, entries 1–3), respectively. The selectivity to cyclohexanone over all three catalysts was above 96 % with cyclohexanol as the only byproduct. The hydrogenation of phenol over CNT‐supported Pd catalysts as a function of time was further studied (Figures 3a and S6b). Compared with Pd/OCNT, Pd/NCNT showed a significantly higher reaction rate and slightly improved selectivity, demonstrating the beneficial effect of N‐doping on catalytic activity because of the enhanced dispersion of Pd NPs. The linear increase of conversion over both catalysts suggests a zero‐order reaction kinetics.

**Table 3:** Catalytic performance of supported Pd catalysts in the hydrogenation of phenol to cyclohexanone.^[a]^

Entry	Catalysts	Substrate	Hydrogen Source	Conversion (%)	Selectivity (%)	
					Cyclohexanone	Cyclohexanol
1	Pd/NCNT	Phenol	H_2_^[b]^	87.9	97.6	2.4
2	Pd/OCNT	Phenol	H_2_^[b]^	54.4	96.1	3.9
3	Pd/AC	Phenol	H_2_^[b]^	50.2	96.2	3.8
4	Pd/NCNT	Phenol	FA	57.2	98.4	1.6
5	Pd/OCNT	Phenol	FA	10.8	99.0	1.0
6	Pd/AC	Phenol	FA	16.7	98.0	2.0
7	Pd/NCNT	Cyclohexanone	FA	3.1	–	100
8	Pd/NCNT	Cyclohexanone	H_2_^[b]^	8.0	–	100
9	Pd/OCNT	Cyclohexanone	FA	1.8	–	100

[a] Reaction conditions: 0.05 mmol phenol, 30 mg catalyst, 60 °C, 3 h, 3 mmol formic acid; [b] 1 bar H_2_.

When FA was employed in the hydrogenation of phenol, the obtained degrees of conversion over the Pd‐based catalysts were 57.2 %, 10.8 %, and 16.7 % for Pd/NCNT, Pd/OCNT, and Pd/AC (Table 3, entries 4–6), respectively. Compared with H_2_, FA led to significantly lower conversion, especially for Pd/OCNT and Pd/AC. The selectivity to cyclohexanone for all catalysts was above 98 %, being slightly higher than those with H_2_. The catalytic performance of Pd/NCNT and Pd/OCNT in phenol hydrogenation with FA as a function of time is presented in Figures 3b and S6a. A clear difference in the reaction rate for Pd/NCNT and Pd/OCNT in FA was observed. In contrast to the linear increase of conversion with H_2_, using Pd/OCNT resulted in an almost constant conversion of ca. 11 % after 3 h with FA and was deactivated afterwards. The deactivation of Pd/OCNT in FA is probably due to the competitive adsorption between FA and phenol, resulting in a high steady‐state formate coverage on Pd NPs, which inhibits the adsorption of phenol on Pd NPs, because formate as a bidentate ligand is much more strongly adsorbed than phenol(ate). By comparison, the deactivation of Pd/NCNT in FA was not observed, and the conversion of phenol proceeded continuously, reaching almost full conversion after 13 h. Since Pd/NCNT and Pd/OCNT exhibit similar Pd particle sizes and mesoporous structures, the higher catalytic activity of Pd/NCNT and the deactivation of Pd/OCNT in FA must be attributed to the presence of nitrogen atoms embedded in the surface of NCNTs. Compared with Pd/OCNT, Pd/NCNT exhibits higher turnover frequency (TOF), suggesting the strong promoting effect of N dopant. Furthermore, the TOF values of our catalysts are comparable with previously reported results using either H_2_ or formic acid (or formate) as the hydrogen source (Table S3).

#### Hydrogenation of cyclohexanone to cyclohexanol

Control experiments of the hydrogenation of cyclohexanone to cyclohexanol were additionally conducted under similar reaction conditions (Table 3, entries 7–9). Compared with the conversion of phenol, drastically lower degrees of cyclohexanone conversion were obtained for all cases. The conversion of cyclohexanone with FA was found to be rather low for both catalysts, amounting to 3.1 % for Pd/NCNT and 1.8 % for Pd/OCNT. By comparison, H_2_ resulted in a higher cyclohexanone conversion of 8 % over Pd/NCNT than FA, suggesting that the hydrogenation of cyclohexanone to cyclohexanol was significantly suppressed in FA because of the stronger adsorption of FA than that of cyclohexanone on Pd NPs.

#### Effect of reaction temperature

**Figure 4:**
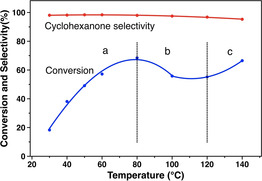
Effect of reaction temperature on the hydrogenation of phenol to cyclohexanone with FA over Pd/NCNT.

The hydrogenation of phenol with FA over Pd/NCNT as a function of reaction temperature was investigated and is shown in Figure 4. The conversion of phenol is enhanced evidently from 18.3 % to 68.5 % when increasing the temperature from 30 °C to 80 °C (region a), but higher temperature led to a slight decrease from 80 °C to 120 °C (region b). Above 120 °C (region c), the degree of phenol conversion to cyclohexanone is significantly enhanced. These temperature‐dependent phenomena are caused by the competition between the two different hydrogenation routes, that is, the transfer hydrogenation with FA and the hydrogenation with in situ formed molecular H_2_ from the decomposition of FA. For example, a separate FA decomposition experiment excluded the formation of H_2_ over Pd/NCNT at 30 °C (Figure 7a, discussed later in detail); nevertheless, FA still resulted in comparable phenol conversion like 5 bar H_2_ (i. e., 18.3 % vs. 19.4 %, Table S4). This observation suggests that only catalytic transfer hydrogenation of phenol with FA occurs at 30 °C. Therefore, we assume that the low‐temperature region a is dominated by the transfer hydrogenation with FA, region b by the competition between FA transfer hydrogenation and H_2_ hydrogenation, and region c by H_2_ hydrogenation and the increasing activity because of the increasing rate constant.

#### Effect of FA/phenol ratio

**Figure 5:**
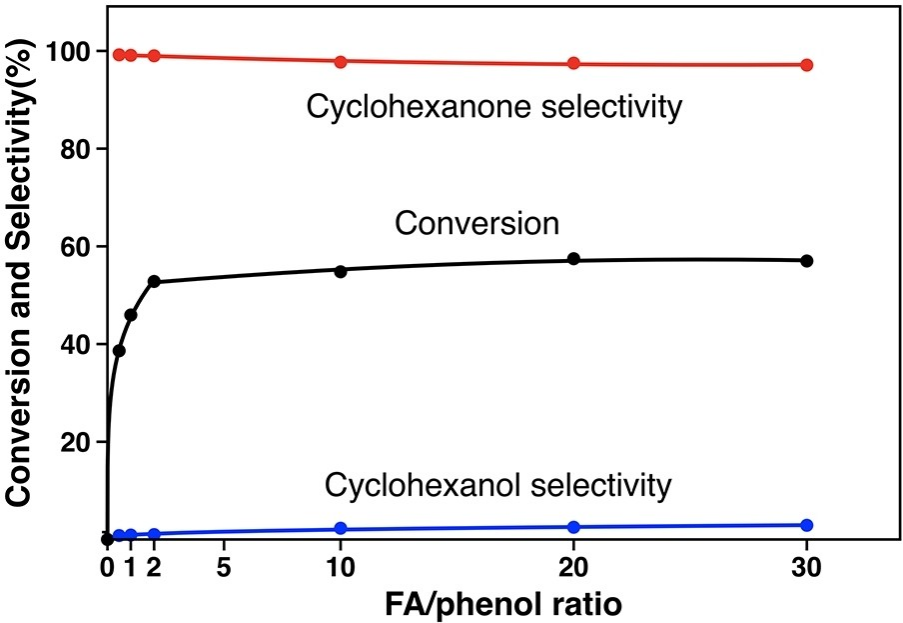
Effect of FA/phenol ratio on the hydrogenation of phenol to cyclohexanone over Pd/NCNT at 60 °C after 3 h.

The effect of the FA/phenol ratio was also studied under similar conditions at 60 °C (Figure 5). Without the addition of FA, no conversion occurred. Upon increasing the FA/phenol ratio to 2, the conversion of phenol strongly increased to 45.8 % and the outstanding selectivity to cyclohexanone was maintained. However, when further increasing the ratio to 30, the degree of phenol conversion only increased slightly. These results show that the rate of phenol hydrogenation strongly depends on the FA amount for low FA/phenol ratios (i. e., ratio <2). The almost constant conversion for FA/phenol ratios higher than 2 suggests that Pd NPs probably are fully covered by formate at higher ratios, and the transfer hydrogenation of phenol with the adsorbed formate is the rate‐determining step.

#### Catalyst reusability

**Figure 6:**
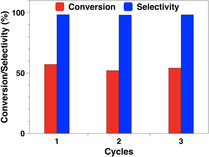
Recycle test of Pd/NCNT in the hydrogenation of phenol to cyclohexanone with FA

The stability and the reusability of Pd/NCNT were also evaluated. The used catalyst kept its high catalytic activity after three runs, and the degree of phenol conversion only slightly decreased from 57.2 % to 54.5 % (Figure 6). Furthermore, the recycled catalyst was characterized by XPS, XRD and STEM (Figures S5, S7 and S8). The N 1s core level XP spectra suggest similar contents of pyridinic N and pyrrolic N in the spent Pd/NCNT as compared to the fresh Pd/NCNT (Figure S5, Table S2). The Pd (111) reflections at 40.2° in the spent catalyst are still hardly visible without significant changes compared with the fresh catalyst. The mean Pd particle size of the fresh and used Pd/NCNT is rather similar amounting to 4.3 and 5.1 nm, respectively. Overall, Pd/NCNT is highly stable in phenol hydrogenation and exhibits excellent reusability.

### Reaction mechanism and deactivation study

#### Deactivation and FA decomposition

As discussed previously, Pd/NCNT and Pd/OCNT exhibited totally different catalytic performances, especially with respect to deactivation in the hydrogenation of phenol with FA. Pd/NCNT was much more active than Pd/OCNT, and Pd/OCNT was deactivated after 3 h while the deactivation of Pd/NCNT was not observed (Figure 3b). As FA decomposition can undergo the two different competing pathways dehydrogenation and dehydration (Scheme 1),[^8^, ^27^] CO formed via the dehydration reaction may result in the deactivation of the catalysts. To this end, FA decomposition over Pd/NCNT and Pd/OCNT was conducted in an autoclave, and the gas product distribution was analyzed to differentiate the dehydrogenation and dehydration pathways. Both catalysts predominantly led to the dehydrogenation of FA to CO_2_, and the yield of CO was found to be 0.13 % for Pd/NCNT and 0.18 % for Pd/OCNT (Table S5). Because of the rather low and similar level of CO concentrations over both catalysts, the deactivation of Pd/OCNT by CO poisoning is excluded.

**Figure 7:**
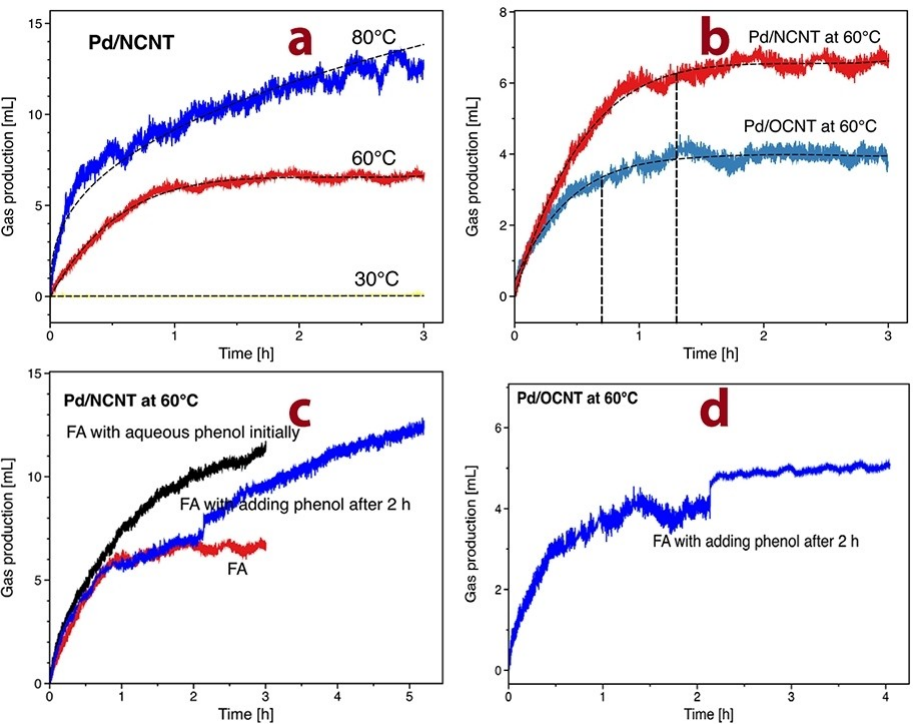
Gas production during FA decomposition over a) Pd/NCNT at 30 °C, 60 °C and 80 °C; b) Pd/NCNT and Pd/OCNT at 60 °C; c) Pd/NCNT at 60 °C without, and with the addition of phenol solution initially or after 2 h; d) Pd/OCNT at 60 °C with the addition of phenol solution after 2 h.

Subsequently, further FA decomposition experiments were performed by monitoring the gas evolution over Pd/NCNT and Pd/OCNT to better understand the catalytic transfer hydrogenation of phenol with FA. Figure 7a shows the gas volume produced during FA decomposition over Pd/NCNT at 30 °C, 60 °C, and 80 °C. At 30 °C, gas production was not detected, indicating that FA decomposition does not occur at such low reaction temperature. FA decomposition proceeded very fast during the first 0.5 h at 60 °C and 80 °C, and then the reaction rate gradually decreased, which even decreased to zero after 1 h at 60 °C. Figure 7b shows that Pd/NCNT and Pd/OCNT led to a similar trend in the FA decomposition at 60 °C, while Pd/NCNT still exhibited a better decomposition activity. This observation is in good agreement with a previous literature report, showing that Pd supported on N‐functionalized mesoporous carbon has a stronger FA decomposition ability than Pd on N‐free carbon.^[28]^ The higher catalytic activity of Pd/NCNT for FA decomposition also partially explains its higher degree of phenol conversion in the hydrogenation of phenol with FA compared with Pd/OCNT. Unlike phenol hydrogenation, where only Pd/OCNT was deactivated, both catalysts suffered from deactivation during the FA decomposition.

Furthermore, FA decomposition was also carried out with the addition of phenol to better understand the different deactivation behavior between FA decomposition and phenol hydrogenation with FA. Pd/NCNT was deactivated in pure FA, whereas such deactivation did not occur in the presence of phenol (Figure 7c). Notably, the deactivated Pd/NCNT catalyst was regenerated by the addition of phenol during FA decomposition (see the blue line in Figure 7c). In contrast, the addition of phenol did not lead to the regeneration of the deactivated Pd/OCNT (Figure 7d).

**Scheme 2:**
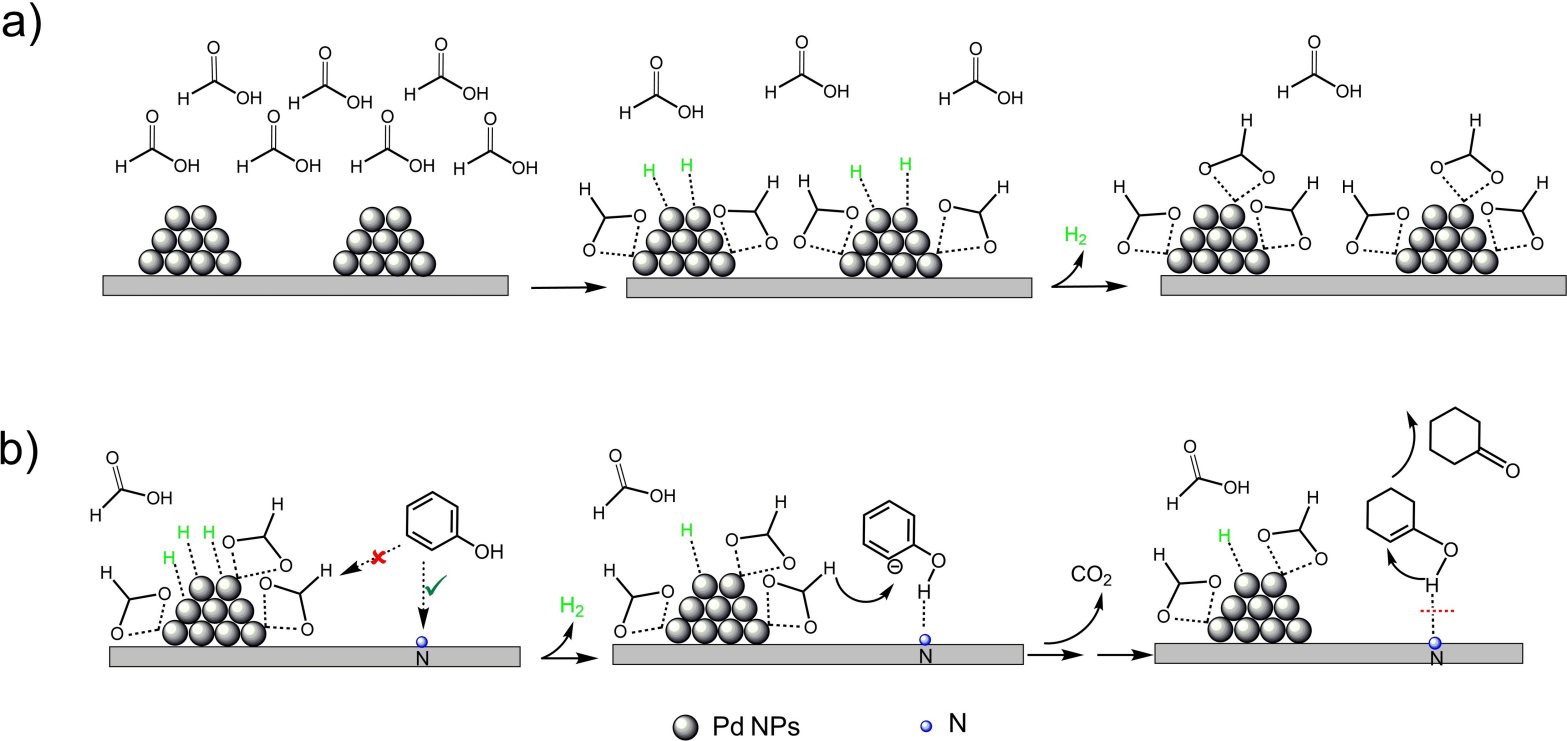
(a) Proposed deactivation mechanism for FA decomposition over CNT‐supported Pd catalysts. (b) Proposed reaction mechanism for the hydrogenation of phenol to cyclohexanone with FA over Pd/NCNT.

Based on these results, we propose that FA decomposition proceeds via the dissociative adsorption of FA to adsorbed formate and hydrogen on the Pd surface. The recombination of two adjacent H adatoms followed by desorption leads to the production of H_2_ initially. With the progress of the reaction, almost all Pd active sites are fully covered by the strongly bound formate, thus suppressing FA decomposition over both Pd/OCNT and Pd/NCNT (Scheme 2a). For the FA decomposition with the addition of phenol and the phenol hydrogenation with FA, phenol can act as hydrogen acceptor favoring the decomposition of adsorbed formate to CO_2_. Since formate as a bidentate ligand is a much stronger adsorbate than phenol(ate), phenol is difficult to be adsorbed on the formate‐covered Pd NPs. Instead, phenol may be adsorbed on the CNTs support, and the excellent resistance of Pd/NCNT towards deactivation compared with Pd/OCNT demonstrates the critical role of N‐doping in the transfer hydrogenation of phenol with FA.

#### Influence of N‐doping

Compared with O‐functionalized CNTs, the N‐doped CNTs can effectively anchor Pd NPs and enhance the dispersion, and thus Pd/NCNT resulted in enhanced catalytic activity in the hydrogenation of phenol with either H_2_ or FA (Table 3). As discussed previously, Pd/NCNT exhibited excellent resistance towards deactivation in the transfer hydrogenation of phenol with FA (Figure 3b) and the reversible deactivation‐regeneration during FA decomposition by adding phenol (Figure 7c), whereas Pd/OCNT was deactivated in both cases. Considering that Pd NPs are almost fully covered by adsorbed formates (Scheme 2a) and the nitrogen functional groups of NCNTs may act as strong Lewis base, the nonplanar adsorption is expected to anchor phenol via weak O−H⋅⋅⋅N interactions (Scheme 2b), which was also proposed in a previous study.^[19b]^ As the surface of OCNTs is more acidic, the adsorption of phenol on OCNTs is probably not favored. Subsequently, the formyl hydrogen of the adsorbed formate reacts with the nonplanarly adsorbed phenolate to produce cyclohexanone after a keto‐enol tautomerism, accompanied by the decomposition of formate to CO_2_ and the release of one free active Pd site (Scheme 2b). Note that it is the formyl hydrogen rather than the hydrogen adatom (originating from the carboxylic acid hydrogen) involved in the transfer hydrogenation, which was proved by isotopic labeling studies.^[29]^

## Conclusion

Pd NPs supported on N‐doped and O‐functionalized carbon nanotubes (Pd/NCNT and Pd/OCNT) were successfully synthesized via the immobilization method and characterized by N_2_ physisorption, XRD, TEM, and XPS. Both catalysts exhibit similar Pd particle sizes, while nitrogen doping modified the surface structure and improved the basicity of Pd/NCNT. Highly selective hydrogenation of phenol to cyclohexanone was achieved over the Pd‐based catalysts under mild conditions using either H_2_ or FA as the hydrogen source. Compared with Pd/OCNT, Pd/NCNT exhibited higher catalytic activity and excellent reusability, especially for the transfer hydrogenation of phenol with FA. It was found that the low‐temperature region (30–80 °C) was dominated by the transfer hydrogenation with FA, the intermediate region (80–120 °C) by the competition between the FA transfer hydrogenation and the H_2_ hydrogenation, and the high‐temperature region (>120 °C) by the H_2_ hydrogenation.

No deactivation of either Pd/OCNT or Pd/NCNT was observed in the hydrogenation of phenol with H_2_. In contrast, Pd/OCNT was deactivated during the transfer hydrogenation with FA, while Pd/NCNT kept its activity during the reaction. Separate FA decomposition experiments without or with the addition of phenol were performed to understand the deactivation mechanism and to unravel the role of N‐doping. Both catalysts were found to be deactivated during FA decomposition. Nevertheless, it was possible to regenerate the deactivated Pd/NCNT by the addition of phenol, but not the deactivated Pd/OCNT. Combining these comparative results, we propose that deactivation is caused by the almost full coverage of active Pd sites by the strongly bound formates, suppressing further FA decomposition and/or transfer hydrogenation of phenol with FA on Pd NPs. Pd/NCNT was not deactivated during the transfer hydrogenation, demonstrating the unique role of N functional groups. NCNTs act as a strong Lewis base and adsorb phenol via special O−H⋅⋅⋅N interactions. Subsequently, the nonplanarly adsorbed phenolate reacts with the adsorbed formate to produce cyclohexanone.

## Experimental Section

**Materials**. All commercially available reagents were used as received without any further purification unless otherwise specified. Palladium chloride (99 %), phenol (≥99.0 %), and formic acid (≥95 %) were obtained from Sigma‐Aldrich. HPLC water and acetonitrile (99.99 %) were supplied by VWR.

Pristine CNTs (diameter about 9 nm, length about 1.5 μm, Nanocyl SA, Belgium) were washed at room temperature in 1.5 M HNO_3_ for 72 h to remove the residual catalysts used for their growth. OCNTs were synthesized by treating the purified CNTs in HNO_3_ vapor at 200 °C for 48 h. A detailed description of the preparation of OCNTs can be found elsewhere.^[30]^ NCNTs were prepared by thermal treatment of OCNTs in 10 vol % NH_3_ at 400 °C.^[31]^ The functionalized OCNTs and NCNTs were dried overnight at 80 °C and ground for further use. Activated carbon (AC, Norit SX2) was obtained from Sigma‐Aldrich as reference material.

**Catalyst preparation**. Pd/NCNT, Pd/OCNT and Pd/AC catalysts were synthesized using a sol‐immobilization method. Briefly, an aqueous solution of PdCl_2_ of the desired concentration was prepared with subsequent addition of a polyvinylalcohol (PVA) solution (1 wt% solution, MW=9000 −10000 g/mol) (PVA/Pd (wt/wt)=1.2). A freshly prepared aqueous NaBH_4_ solution (0.1 M) was then added, forming a dark‐brown colloidal solution containing the Pd nanoparticles. After 30 min of sol generation, the powder carbon support was added into the dark‐brown colloidal solution with a proper pH under vigorous stirring. After 2 h, the solid catalyst was obtained by filtration and washing with distilled water. The catalyst was dried overnight at 80 °C. The required amount of carbon material was calculated to achieve a nominal metal loading of 1 wt%.

**Characterization**. XRD patterns were recorded with a PANalytical X'Pert Pro diffractometer. As an X‐ray source, Ni‐filtered Cu Kα radiation (40 kV, 40 mA) without a monochromator was used. The diffraction patterns were obtained by scanning from 5° to 80° 2θ. Scanning transmission electron microscopy (STEM) and transmission electron microscopy (TEM) measurements were carried out using a JEOL JEM‐2800 with an acceleration voltage of 200 kV. The specimens for STEM and TEM were prepared by ultrasonically dispersing the powder samples in high‐purity ethanol and then allowing a drop of the suspension to evaporate on a copper grid coated with carbon. Pd loading was determined by atomic absorption spectroscopy (AAS) with a PerkinElmer AAS Model Analyst200 after acid digestion.

X‐ray photoelectron spectroscopy (XPS) measurements were performed in an ultrahigh vacuum setup equipped with a high‐resolution data Gamma Scienta SES 2002 analyzer. A monochromatic Al Kα X‐ray source (1486.6 eV, anode operating at 14.5 kV and 30.5 mA) was used as incident radiation. The pressure inside the measuring chamber was kept in the range of 3.5 to 7×10^−10^ mbar during the measurement. The analyzer width was set at 0.3 mm, and the pass energy was fixed at 200 eV for all measurements, resulting in an overall energy resolution better than 0.5 eV. Charging effects were mediated by using a flood gun (SPECS). All spectra were calibrated based on the C 1s binding energy of 284.5 eV. The CASA XPS program with a mixed Gaussian‐Lorentzian function and Shirley background subtraction was employed in the analysis of the XPS data.

### Hydrogenation of phenol to cyclohexanone

**Phenol hydrogenation with FA**. A 12 mL Q‐tube^TM^ glass reactor (Sigma, maximum 120 psi) was used to perform the liquid‐phase hydrogenation of phenol with FA. Typically, 30 mg powder catalysts and 3.0 mmol formic acid were mixed with 2 mL 0.025 M of aqueous phenol solution in the Q‐tube glass reactor. The reactor was purged five times with N_2_ and then pressurized with N_2_ to 1 bar. The reaction was conducted at 60 °C for 1, 2, 3, 4, 5, 6, 7 or 12 h. The stirring speed was set at 500 rpm. The liquid samples diluted with HPLC water were filtered using membrane filters and analyzed by gas chromatography (GC). GC analysis was carried out using an Agilent 7820A GC system with a DB‐WAX column (30 m×0.25 mm×0.25 μm) and an FID detector. The main product is cyclohexanone, with cyclohexanol as the only byproduct.

**Phenol hydrogenation with H_2_**. A 12 mL Q‐tube^TM^ gas purging reactor (Sigma, maximum 180 psi) was used to perform the liquid‐phase hydrogenation of phenol with H_2_. The Q‐tube^TM^ gas purging set includes a normal Q‐tube glass reactor, a connecting hose, a pressure gauge, and two valves, which were used for gas transfer. Typically, 30 mg catalysts were mixed with 2 mL 0.025 M of aqueous phenol solution in the Q‐tube glass reactor. The reactor was purged five times with H_2_ and then pressurized with H_2_ to 1 bar. The reaction was conducted at 60 °C for 1, 2, 3, 4 or 5 h. The stirring speed was set at 500 rpm. The liquid samples diluted with HPLC water were filtered using membrane filters and analyzed by GC.

**Reusability study**. The spent Pd/NCNT catalyst was recovered from the resulting reaction mixture by centrifugation, washing with water and ethanol, and then drying overnight at 80 °C. The recovered Pd/NCNT catalyst was subsequently reused for the hydrogenation of phenol under the standard condition in the presence of FA. After 3 h, the sample was taken and analyzed by GC.

**FA decomposition**. A gas meter setup (Figure S1, Gasmess‐5, MesSen Nord GmbH) was used to carry out FA decomposition by monitoring the gas evolution. The reactor was purged three times with He and then pressurized with 1 bar He. Typically, 30 mg powdery catalysts were mixed with 2 mL water in the glass reactor. After heating to the desired temperature, 3 mmol FA was added into the solution. The magnetic stirrer was set at 500 rpm. The gas evolution was continuously monitored at 30 °C, 60 °C, or 80 °C for 3 h.

## Conflict of interest

The authors declare no conflict of interest.

## Supporting information

As a service to our authors and readers, this journal provides supporting information supplied by the authors. Such materials are peer reviewed and may be re‐organized for online delivery, but are not copy‐edited or typeset. Technical support issues arising from supporting information (other than missing files) should be addressed to the authors.

Supporting Information
