# Supplementary material for: The Role of Nitrogen‐doping in the Catalytic Transfer Hydrogenation of Phenol to Cyclohexanone with Formic Acid over Pd supported on Carbon Nanotubes
Source: Chemistry. 2021 Jun 18;27(42):10948–56. doi: 10.1002/chem.202100981 (PMC8361974; doi:10.1002/chem.202100981)
Supplement: Supplementary file 1 — Supporting Information [file CHEM-27-10948-s001.pdf]

Chemistry–A European Journal

Supporting Information

The Role of Nitrogen-doping in the Catalytic Transfer Hydrogenation of Phenol to Cyclohexanone with Formic Acid over Pd supported on Carbon Nanotubes

Bin Hu, Xiaoyu Li, Wilma Busser, Stefan Schmidt, Wei Xia, Guangci Li, Xuebing Li, and Baoxiang Peng*

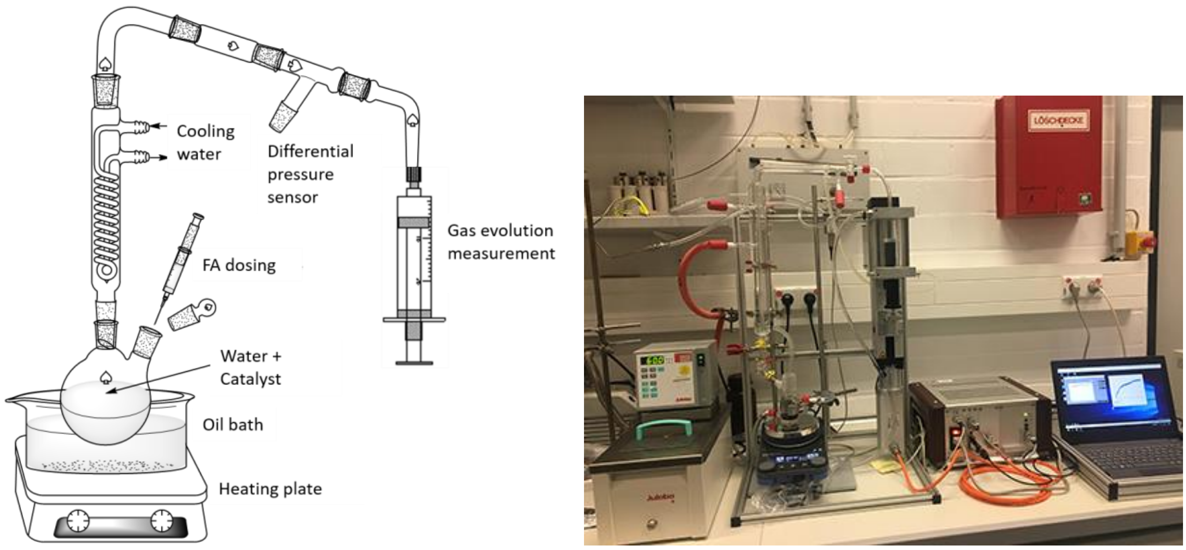

Figure S1. Schematic diagram (left) and a photo (right) of the gas-meter setup (Gasmess-5, MesSen Nord GmbH) used for the decomposition of formic acid.

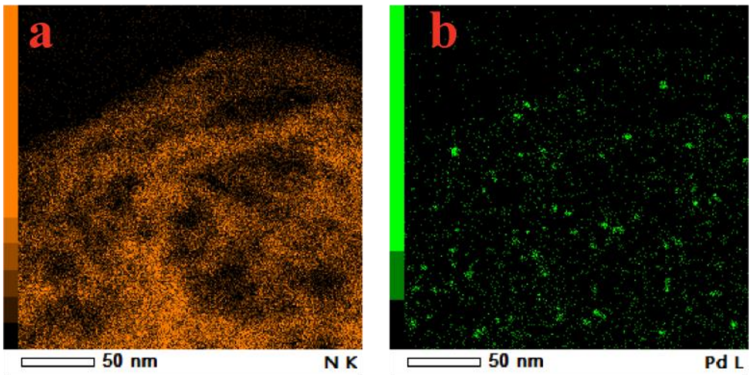

Figure S2. EDS elemental mapping of N and Pd in Pd/OCNT

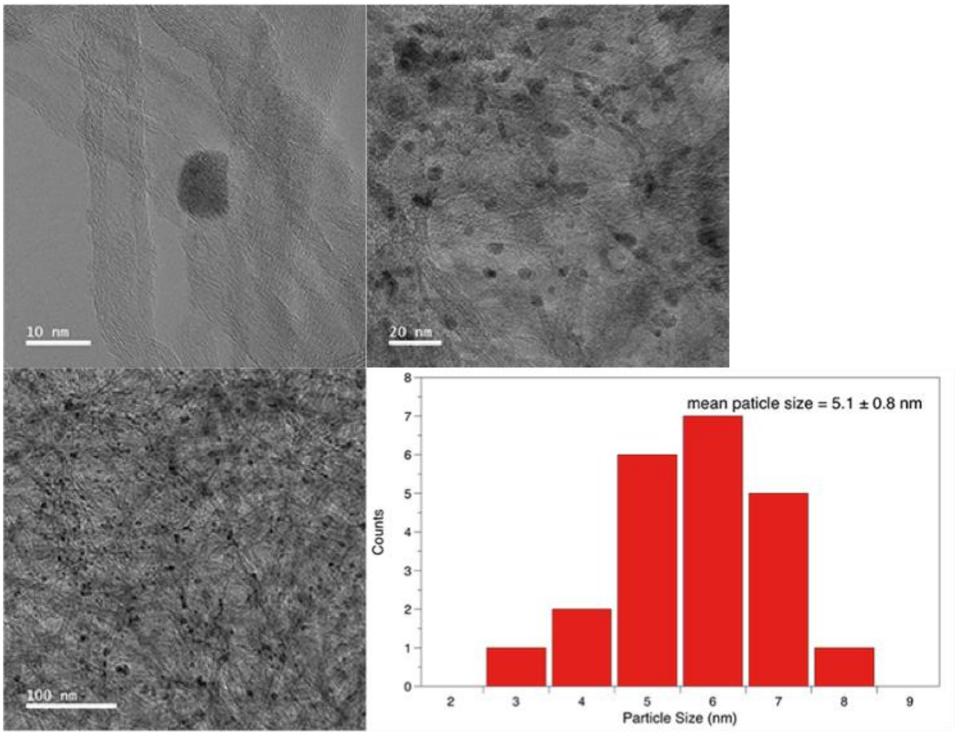

Figure S3. TEM images and particle size distribution of Pd/OCNT.

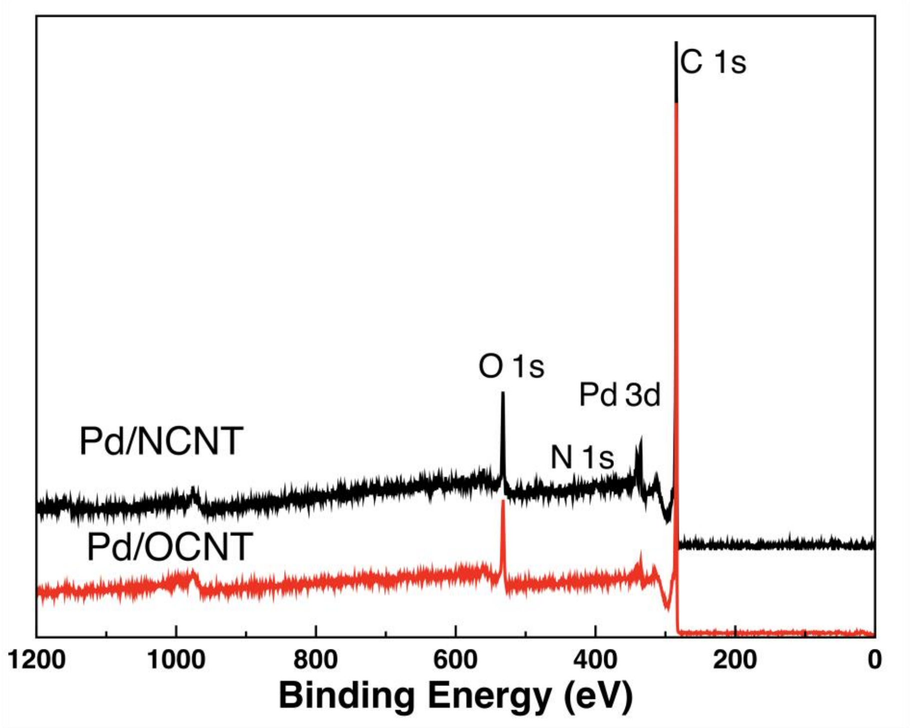

Figure S4. XPS survey spectra of Pd/NCNT and Pd/OCNT.

Table S1. Relative abundance (%) of the components determined from the Pd $3d_{5/2}$ spectra.

Sample	Component/binding energy (eV)	
	Pd^0	PdO
	335.3	337.0
Pd/NCNT	61	39
Pd/OCNT	45	55

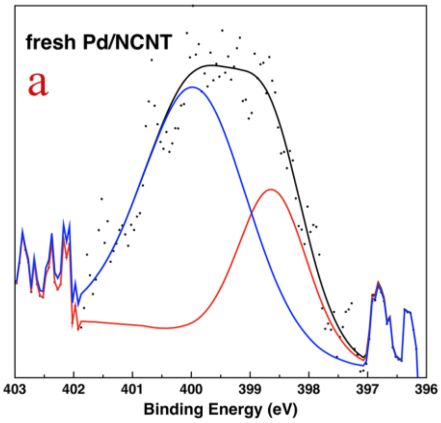

Figure S5. N 1s region of the XPS results for a) fresh Pd/NCNT and b) spent Pd/NCNT.

Table S2. Relative abundance (%) of the components determined from the N 1s spectra.

Sample	Component/binding energy (eV)	
	Pyridinc N	Pyrrolic N
	398.6	400.2
Pd/NCNT-fresh	68	32
Pd/NCNT-spent	70	30

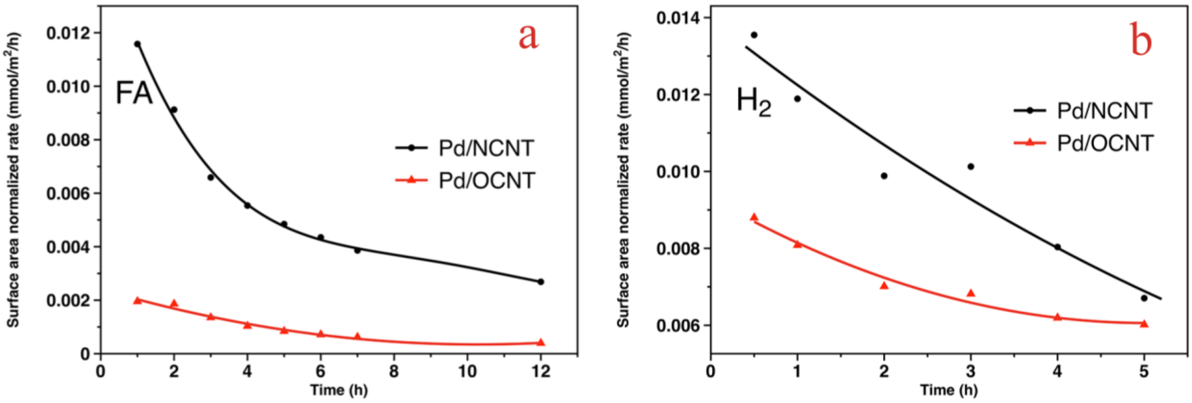

Figure S6. Surface area normalized reaction rates for Pd/NCNT and Pd/OCNT as a function of time in the presence of (a) FA and (b) H_2.

Table S3. Catalytic activity of different catalysts in phenol hydrogenation

Entry	Catalysts	Hydrogen Source	Time (h)	Pressure (bar)	T (°C)	Solvent	Conversion (mol %)	$S_{cyclohexanone}$ (mol %)	TON	TOF (h^{-1})	Ref.
1	Pd/NCNT[a]	H_2	3	1	60	H_2O	87.9	97.6	15.5	5.2	Tw
2	Pd/NCNT[a]	FA	3	1	60	H_2O	57.2	98.4	10.1	3.4	Tw
3	Pd/OCNT[a]	H_2	3	1	60	H_2O	54.4	96.1	9.4	3.1	Tw
4	Pd/OCNT[a]	FA	3	1	60	H_2O	10.8	99.0	1.7	0.6	Tw
5	Pd/mpg-C_3N_4	H_2	2	1	65	H_2O	99.0	99.0	20	10	[1]
6	Pd/Al-MIL-53-NH_3	H_2	5	5	50	H_2O	90.6	97.0	-	16	[2]
7	Pd/Al-MIL-101	H_2	2	5	50	H_2O	85.0	98.8	-	52	[3]
8	Pd/C	HCOONa	0.25	-	80	H_2O	100	98.0	-	28	[4]
9	Pd-PANI/CNT	H_2	9	1	80	H_2O	99.0	99.0	-	0.9	[5]
10	Pd@CN-TiO_2-25	H_2	1.2	1	80	H_2O	98.0	98.0	-	13	[6]
11	Pd/C (commercial)	FA	4	3 (N_2)	50	H_2O	56	98.6	29.3	7.3	[7]
12	Pd/AC	FA	4	3 (N_2)	50	H_2O	65.6	96.3	39.1	9.8	[7]
13	Pd/MIL-101	FA	4	3 (N_2)	50	H_2O	37.9	98.2	20.1	5.0	[7]
14	Pd/TiO_2	FA	4	3 (N_2)	50	H_2O	24.7	99.2	10.3	2.6	[7]
15	Pd/AC	HCOOK	6	-	90	H_2O	99	99	-	8.3	[8]
16	Pd/AC	FA	6	-	90	H_2O	19	99	-	1.6	[8]
17	Pd/AC	H_2	6	1	90	H_2O	100	78	-	6.5	[8]

[a] Reaction conditions: 0.05 mmol phenol, 30 mg catalyst, 60 °C, 3 mmol formic acid, or 1 bar H_2.

Table S4. Comparison of phenol hydrogenation with FA and H_2 over Pd/NCNT at 30 °C after 3 h

Hydrogen source	Conversion (%)	Selectivity (%)	
		Cyclohexanone	Cyclohexanol
3 mmol FA*	18.3	98.1	1.9
1 bar H_2	10.7	97.4	2.6
5 bar H_2	19.4	96.7	3.3

* Note that if the 3 mmol FA is fully decomposed to CO_2 and H_2 in the reactor, the partial pressure of H_2 is calculated to be about 5 bar.

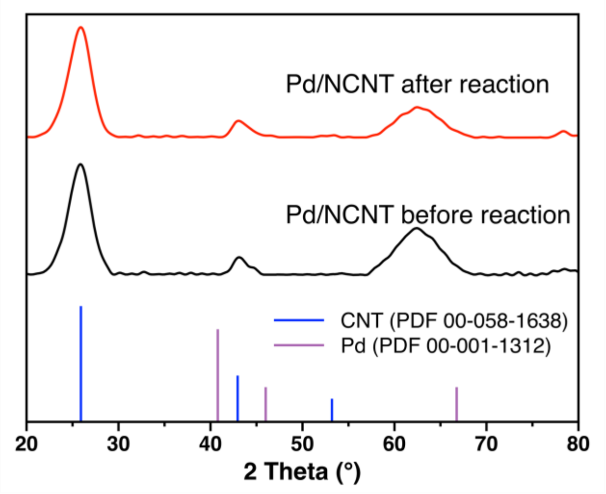

Figure S7. XRD patterns of Pd/NCNT before and after reaction.

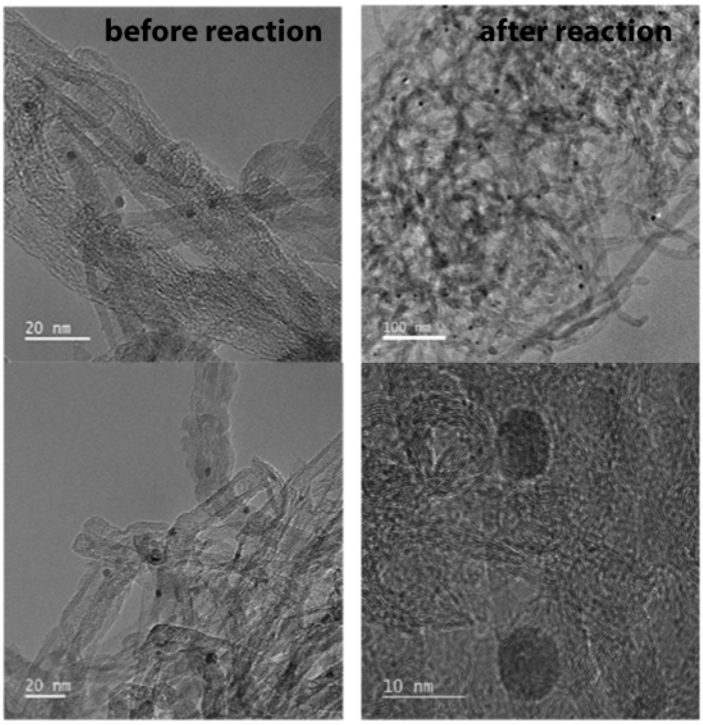

Figure S8. TEM images of Pd/NCNT before and after reaction.

Table S5. Gas distribution during FA decomposition over Pd/NCNT and Pd/OCNT at 60 °C

Sample	Yield	
	CO_2	CO
Pd/NCNT	15.9%	0.13%
Pd/OCNT	12.5%	0.18%

Reaction conditions: 50 mg catalyst, 15 mmol fomic acid, 10 mL water, 60 °C, 5 bar N_2, 3 h

References:

[1] Wang Y., Yao J., Li H., et al. Highly Selective Hydrogenation of Phenol and Derivatives over a Pd@Carbon Nitride Catalyst in Aqueous Media [J]. ***J. Am. Chem. Soc.***, 2011, 133(8): 2362-2365.

[2] Zhang D., Guan Y., Hensen E. J. M., et al. Tuning the Hydrogenation Activity of Pd Nps on Al–Mil-53 by Linker Modification [J]. ***Catal. Sci. Technol.***, 2014, 4(3): 795-802.

[3] Zhang D., Guan Y., Hensen E. J. M., et al. Porous Mofs Supported Palladium Catalysts for Phenol Hydrogenation: A Comparative Study on Mil-101 and Mil-53 [J]. ***Catal. Commun.***, 2013, 41: 47-51.

[4] Cheng H., Liu R., Wang Q., et al. Selective Reduction of Phenol Derivatives to Cyclohexanones in Water under Microwave Irradiation [J]. ***New J. Chem.***, 2012, 36(4): 1085-1090.

[5] Chen J., Zhang W., Chen L., et al. Direct Selective Hydrogenation of Phenol and Derivatives over Polyaniline-Functionalized Carbon-Nanotube-Supported Palladium [J]. ***ChemPlusChem***, 2013, 78(2): 142-148.

[6] Ding S., Zhang C., Liu Y., et al. Pd Nanoparticles Supported on N-Doped Porous Carbons Derived from Zif-67: Enhanced Catalytic Performance in Phenol Hydrogenation [J]. ***J. Ind. Eng. Chem.***, 2017, 46: 258-265.

[7] Zhang D., Ye F., Xue T., et al. Transfer Hydrogenation of Phenol on Supported Pd Catalysts Using Formic Acid as an Alternative Hydrogen Source [J]. ***Catal. Today***, 2014, 234, 133-138.

[8] Patil R. D., Sasson Y. Selective Transfer Hydrogenation of Phenol to Cyclohexanone on Supported Palladium Catalyst Using Potassium Formate as Hydrogen Source under Open Atmosphere [J]. ***Applied Catalysis A: General***, 2015, 499, 227-231.
